# Visceral Adipose Predicts Prognosis and Toxicities in Locally Advanced Bladder Cancer Patients Treated With Adjuvant Gemcitabine Plus Cisplatin Chemotherapy

**DOI:** 10.1002/cam4.70742

**Published:** 2025-03-27

**Authors:** Zhimin Gao, Lei Zhang, Zhen Li, Xu Qin, Zewei Wang, Junqi Wang, Nienie Qi, Hailong Li

**Affiliations:** ^1^ Department of Urology The Affiliated Hospital of Xuzhou Medical University Xuzhou China; ^2^ Graduate School of Xuzhou Medical University Xuzhou China

**Keywords:** bladder cancer, body composition, cisplatin, gemcitabine, prognosis, risk factors, urothelial carcinoma, visceral adipose

## Abstract

**Purpose:**

For patients with bladder cancer (BC) undergoing radical cystectomy followed by adjuvant chemotherapy, the impact of visceral adipose tissue on prognosis and chemotherapy‐related toxicities has not been well established.

**Materials and Methods:**

From July 2013 to November 2020, 224 BC patients received adjuvant gemcitabine plus cisplatin at our institution. Computed tomography images of the patients were analyzed to calculate the visceral adipose tissue index (VATI). Patients were stratified into high‐ and low‐VATI groups based on a predetermined cutoff value, and differences in prognosis and chemotherapy‐related adverse events (AEs) between the two groups were compared.

**Results:**

After propensity score matching, a total of 166 patients were enrolled, with 83 in the low‐VATI group and 83 in the high‐VATI group. The low‐VATI group exhibited notably extended progression‐free survival (PFS) in comparison to the high‐VATI group (*p* = 0.044). Conversely, no substantial variation was noted concerning overall survival (OS) among the patient cohorts. In the multivariable Cox regression analysis, patients aged over 70 years (HR = 1.66, 95% CI 1.09–2.57, *p* = 0.04) and nodal positivity (HR = 2.98, 95% CI 1.04–4.28, *p* = 0.01) emerged as significant risk factors for OS. In addition to the level of VATI (HR = 2.47, 95% CI 1.02–4.21, *p* = 0.04), nodal positivity (HR = 4.04, 95% CI 1.30–12.56, *p* = 0.02) remained a significant risk factor for PFS. Regarding chemotherapy‐related AEs, the most common AEs of any grade and grade ≥ 3 were hematologic toxicities. Patients in the low‐VATI group exhibited a higher likelihood of experiencing grade ≥ 3 neutropenia compared to those in the high‐VATI group (*p* = 0.04).

**Conclusions:**

This study demonstrated that, among patients treated with adjuvant chemotherapy for locally advanced BC, patients in the low‐VATI group exhibited a significantly prolonged PFS compared to those in the high‐VATI group. However, no significant difference was observed in terms of OS. Regarding chemotherapy‐related AEs, patients in the high‐VATI group exhibited a relatively lower incidence and severity of toxic reactions.

## 1 Introduction

There is an increasing variety of treatment options regarding locally advanced or metastatic urothelial carcinoma (UC), such as immune checkpoint inhibitors (ICIs) [1], antibody–drug conjugates (ADCs) [2], and fibroblast growth factor receptor (FGFR) inhibitors [3]. Nevertheless, the standard initial therapeutic approach for advanced bladder cancer (BC) still involves combination chemotherapy based on cisplatin [4]. The efficacy of gemcitabine plus cisplatin (GC) as neoadjuvant chemotherapy (NAC) [5] and adjuvant chemotherapy (AC) [6] in BC patients has been demonstrated.

The body mass index (BMI) serves as a straightforward measure, employing height and weight, frequently utilized for the categorization of overweight and obesity in humans. Research findings have demonstrated a robust association between BMI and postoperative complications [7] as well as prognosis [8] in patients with UC. With the deepening of the research, there is a growing acknowledgment of muscle and adipose tissue functioning as distinct organs [9]. Hence, the definition of obesity based on BMI may be misleading [10], given that individuals with identical BMI values may exhibit diverse body compositions. In the human body, adipose tissue comprises predominantly two components: subcutaneous adipose tissue (SAT) and visceral adipose tissue (VAT). These two different types of adipose tissue have been demonstrated to serve distinct functions [11], playing pivotal and unique roles in the prognosis of UC [12, 13, 14]. Nevertheless, there is currently no consensus regarding the impact of VAT on the prognosis and chemotherapy‐related toxicities in patients with BC after receiving first‐line AC.

This study aimed to elucidate the influence of VAT on the prognosis and the treatment‐related adverse events (TRAEs) in patients receiving postoperative chemotherapy for locally advanced BC.

## 2 Materials and Methods

### 2.1 Patients Characteristics

From July 2013 to November 2020, we retrospectively evaluated 224 patients with BC who received postoperative chemotherapy after radical cystectomy (RC) at our institution. Eligible patients had histopathologically confirmed locally advanced bladder urothelial carcinoma (pathologic T3‐4 and/or nodal involvement) and received GC as postoperative AC within 3 months. Sufficient hematological assessments were necessary, encompassing baseline hematological information taken within 7 days prior to the initiation of GC treatment, along with routine postchemotherapy blood tests. In addition, the date of follow‐up and causes of death were collected.

Patients without available preoperative computed tomography (CT) scanning were excluded. Patients who received systemic steroids or other drugs to promote cell proliferation within 1 month before the initiation of GC should also be excluded. All patients provided written informed consent, and the research protocol received approval from the Ethics Committee of the Affiliated Hospital of Xuzhou Medical University.

### 2.2 Treatment and Data Collection

Patients received 21‐day cycles of gemcitabine (administered intravenously on days 1 and 8 of each cycle at a dose of 1000 mg/m^2^ body surface area) and cisplatin (administered intravenously on day 2 of each cycle at a dose of 70 mg/m^2^ body surface area). Patients were administered four cycles of AC unless there was evidence of disease progression, the occurrence of unacceptable toxicity, or a request for discontinuation.

Clinicopathological data were collected, including age, sex, BMI, smoking history, hypertension, diabetes, hydronephrosis, Eastern Cooperative Oncology Group performance score (ECOG‐PS), pathologic tumor stage, and nodal status. Relevant laboratory data, including hematological data, were collected before the initiation of GC and after each chemotherapy cycle. In detail, laboratory parameters include neutrophil count, white blood cell (WBC) count, hemoglobin, and platelet count. Patients with renal insufficiency need to meet both of the following requirements: blood creatinine > 133 μmol/L and eGFR > 60 mL/min. The incidence and grade of TRAEs were assessed using the National Cancer Institute's Common Terminology Criteria for Adverse Events (CTCAE) Version 4.03.

### 2.3 Measurement of Visceral Adipose

CT scan has been confirmed to provide cross‐sectional information about skeletal muscle and adipose tissue, facilitating a more precise and detailed assessment of body compositions by clinicians [15]. We chose the third lumbar vertebra (L3) section from the CT images as the measurement plane, as previous research has demonstrated a linear correlation between the VAT area measured at the L3 level and the total VAT volume [16]. Hence, the L3 level can be regarded as the optimal site for the measurement of visceral adipose. The quantification of VAT area was performed using Osirix DICOM Viewer version 12, with Hounsfield unit (HU) references set at −150 to −50 HU for the assessment of visceral adipose [17]. The Grow Region tool was used to distinguish between different types of tissue. The VAT index (VATI) was calculated by averaging the VAT area measured on two consecutive CT images at the L3 level and then dividing it by the square of one's height in meters.

At present, the optimal cutoff value for VATI is not clear. Consequently, we applied the methodology used in a previous study to determine the cutoff value [18]. Sex‐specific optimal cutoff values were used to dichotomize patients into high‐ or low‐VATI groups, with overall survival (OS) as the primary outcome. The optimal cutoff for VATI was determined using the Receiver Operating Characteristic (ROC) curve, and the selection of the best cutoff value was based on the Yoden index. For male patients with locally advanced BC, the VATI value < 35.2 cm^2^/m^2^ was classified into the low‐VATI group, while for females, the VATI value < 44.7 cm^2^/m^2^ was categorized as the low‐VATI group.

### 2.4 Statistical Analyses

The study's primary endpoint was OS, measured as the duration from the date of cystectomy to the date of death from any cause or the latest available information for patients still alive. The secondary endpoints included progression‐free survival (PFS) as well as the incidence and severity of adverse events (AEs) related to chemotherapy. PFS was defined as the interval between the date of cystectomy and the date of progression or death from any cause.

To reduce the impact of confounding factors, the propensity score matching (PSM) method was employed to match patients with high VATI to those with low VATI. For each patient, the propensity score was calculated to match the following variables: age, sex, BMI, smoking history, hypertension, diabetes, hydronephrosis, ECOG‐PS, pathologic tumor stage, and renal function. Differences between the pre‐ and postmatching groups were assessed utilizing the chi‐square test. PSM was conducted through a one‐to‐one matching approach, and the predefined caliper was implemented to ensure that the standardized difference in all confounding factors remained < 0.2.

The Kaplan–Meier method was employed to estimate OS and PFS, and differences between the survival curves were assessed using the log‐rank test. The ROC curve was used to determine the optimal cutoff for VATI, and the best cutoff value was calculated according to the Yoden index. Univariable and multivariable Cox proportional hazards models were employed to calculate hazard ratios (HRs) and corresponding 95% confidence intervals (CIs). In the univariate analyses, variables with a significance level of *p* < 0.05, along with the pertinent variable “VATI” in this study, were included in the subsequent multivariate analysis. Statistical analysis was performed using SPSS 26.0 (IBM Corp., NY, USA), while PSM was executed using R version 4.0.5 (R Foundation for Statistical Computing, Vienna, Austria). All statistical analyses were two‐sided, with statistical significance set at *p* < 0.05.

## 3 Results

Before PSM, a total of 224 BC patients who had received first‐line adjuvant GC were included in this study, including 109 patients with high VATI and 115 with low VATI. Differences were statistically significant in terms of sex, BMI, the presence of diabetes, and ECOG‐PS (Table 1). Compared with patients in the high‐VATI group, those in the low‐VATI group had a higher proportion of females (High VATI 38.5%; Low VATI 54.8%), and a greater number of patients had a BMI < 25 kg/m^2^ (High VATI 51.4%; Low VATI 64.3%).

**TABLE 1:** Patient demographics and baseline disease characteristics.

Variables, *n* (%)	Before PSM		After PSM		Standardized difference	
	High VATI (*n* = 109)	Low VATI (*n* = 115)	High VATI (*n* = 83)	Low VATI (*N* = 83)	Pre‐PSM	Post‐PSM
Median age [range], years	65 [33–84]	62 [34–84]	64 [35–81]	61 [34–83]		
Age group						
≤ 70	82 (75.2)	77 (67.0)	57 (68.7)	50 (60.2)	0.173	0.256
> 70	27 (24.8)	38 (33.0)	26 (31.3)	33 (39.8)		
Sex						
Female	42 (38.5)	63 (54.8)	31 (37.3)	34 (41.0)	0.015	0.633
Male	67 (61.5)	52 (45.2)	52 (62.7)	49 (59.0)		
BMI						
< 25	56 (51.4)	74 (64.3)	38 (45.8)	43 (51.8)	0.049	0.438
≥ 25	53 (48.6)	41 (35.7)	45 (54.2)	40 (48.2)		
Smoking history						
No	38 (34.9)	44 (38.3)	37 (44.6)	41 (49.4)	0.598	0.534
Yes	71 (65.1)	71 (61.7)	46 (55.4)	42 (50.6)		
Hypertension						
No	90 (82.6)	89 (77.4)	66 (79.5)	61 (73.5)	0.334	0.360
Yes	19 (17.4)	26 (22.6)	17 (20.5)	22 (26.5)		
Diabetes						
No	86 (78.9)	107 (93.0)	72 (86.7)	75 (90.4)	0.002	0.465
Yes	23 (21.1)	8 (7.0)	11 (13.3)	8 (9.6)		
Hydronephrosis						
No	69 (63.3)	68 (59.1)	45 (54.2)	42 (50.6)	0.522	0.641
Yes	40 (36.7)	47 (40.9)	38 (45.8)	41 (49.4)		
ECOG status						
0	83 (76.1)	73 (63.5)	60 (72.3)	56 (67.5)	0.039	0.499
1	26 (23.9)	42 (36.5)	23 (27.7)	27 (32.5)		
Pathologic tumor stage/nodal status						
T3/N−	47 (43.1)	59 (51.3)	41 (49.4)	48 (57.8)	0.237	0.310
T4/N−	11 (10.1)	17 (14.8)	11 (13.2)	15 (18.1)		
Nx	15 (13.8)	10 (8.7)	13 (15.7)	9 (10.8)		
N+	36 (33.0)	29 (25.2)	18 (21.7)	11 (13.3)		
Renal dysfunction						
No	95 (87.2)	96 (83.5)	69 (83.1)	69 (83.1)	0.438	1.000
Yes	14 (12.8)	19 (16.5)	14 (16.9)	14 (16.9)		

Abbreviations: BMI, body mass index; ECOG, Eastern Cooperative Oncology Group; PSM, propensity score matching; VATI, visceral adipose tissue index.

After PSM, data for 166 patients were available for analysis (83 in each group). No significant difference was found between the two groups (Table 1). In both groups, the majority of BC patients undergoing RC were under the age of 70 (High VATI 68.7%; Low VATI 60.2%) and had an ECOG score of 0 (High VATI 72.3%; Low VATI 67.5%).

### 3.1 Patient Survival

**FIGURE 1:**
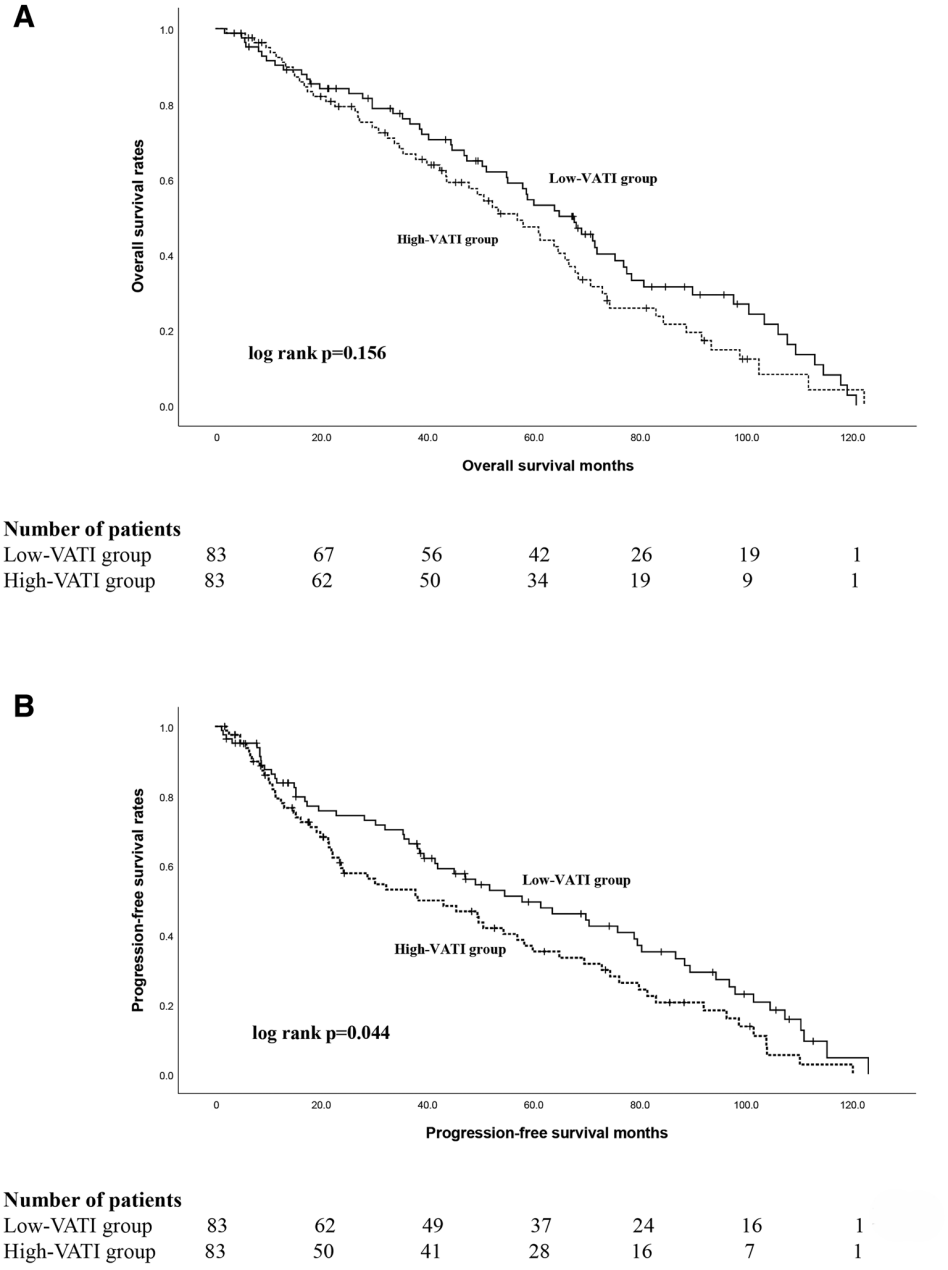
Kaplan–Meier survival curves in patients with high‐ and low‐VATI groups. Kaplan–Meier curves were plotted to estimate overall survival (A) and progression‐free survival (B). The log‐rank test was used to compare the differences between groups.

The median duration of follow‐up for all patients was 63.5 months. The median OS of patients was 67.5 (95% CI 55.8–79.2) months in the low‐VATI group and 56.8 (95% CI 45.1–68.5) months in the high‐VATI group. The 5‐year OS rate for the low‐VATI group was 50.6%, while the high‐VATI group exhibited a rate of 41.0% over the same periods (Figure 1A). Although the level of VATI was not found to be significantly associated with differences in OS (*p* = 0.156), a trend toward a protective effect was observed in BC patients undergoing adjuvant GC chemotherapy.

The low‐VATI group exhibited significantly longer PFS compared to the high‐VATI group (*p* = 0.044). The median PFS in the low‐VATI group was 52.8 (95% CI 35.2–80.4) months, which was longer than that of the high‐VATI group, 38.1 (95% CI 18.6–57.6) months (Figure 1B). In addition, the low‐VATI group exhibited a 5‐year PFS rate of 44.6%, compared to a rate of 33.7% observed in the high‐VATI group (Figure 1B). As of November 2023, 37 (22.3%) patients were still alive (High VATI, 17 patients; Low VATI, 20 patients). Among them, 8 (47.1%) of the patients in the high‐VATI group and 11 (55.0%) in the low‐VATI group were still in complete remission, respectively.

### 3.2 Impact of Prognostic Factors on Survival

The influence of various predictors of OS and PFS is delineated in Tables 2 and 3. The level of VATI was not significantly associated with OS, but we observed a certain trend toward significance (*p* = 0.09). In the multivariate analysis of OS, age (HR = 1.66, 95% CI 1.09–2.57, *p* = 0.04) and nodal status (HR = 2.98, 95% CI 1.04–4.28, *p* = 0.01) remained significant risk factors of OS. The HR for patients with high VATI was 1.33 (95% CI 0.87–3.01, *p* = 0.17) compared to that of patients with low VATI.

**TABLE 2:** Univariate and multivariate Cox regression models describing the impact of the studied predictors for OS after receiving postoperative GC.

Variables	Univariate analysis			Multivariate analysis		
	HR	95% CI	*p*	HR	95% CI	*p*
Age						
≤ 70	1			1		
> 70	1.90	0.67–3.81	0.03*	1.66	1.09–2.57	0.04*
BMI						
< 25	1					
≥ 25	0.94	0.53–1.68	0.31			
Smoking history						
Never	1					
Ever	0.82	0.45–1.51	0.46			
Hydronephrosis						
No	1					
Yes	1.1	0.58–1.94	0.86			
ECOG status						
0	1					
1	1.40	0.79–2.48	0.26			
Pathologic tumor stage/nodal status						
T3/N−	1			1		
T4/N−	1.83	0.79–4.37	0.35	1.12	0.68–2.17	0.75
Nx	1.92	0.83–4.62	0.13	1.38	0.77–2.83	0.59
N+	3.32	1.11–9.21	0.02*	2.98	1.04–4.28	0.01*
VATI						
Low VATI	1			1		
High VATI	1.74	0.91–3.33	0.09	1.33	0.87–3.01	0.17
Renal dysfunction						
No	1					
Yes	0.75	0.42–1.33	0.32			

*Note*: *Indicates statistical significance.

Abbreviations: BMI, body mass index; CI, confidence interval, ECOG, Eastern Cooperative Oncology Group; GC, gemcitabine plus cisplatin; HR, Hazard ratio; OS, overall survival; VATI, visceral adipose tissue index.

**TABLE 3:** Univariate and multivariate Cox regression models describing the impact of the studied predictors for PFS after receiving postoperative GC.

Variables	Univariate analysis			Multivariate analysis		
	HR	95% CI	*p*	HR	95% CI	*p*
Age						
≤ 70	1					
> 70	1.11	0.57–2.15	0.77			
BMI						
< 25	1					
≥ 25	0.96	0.53–1.74	0.89			
Smoking history						
Never	1					
Ever	0.87	0.36–1.77	0.49			
Hydronephrosis						
No	1					
Yes	0.94	0.51–1.73	0.84			
ECOG status						
0	1					
1	0.76	0.39–1.48	0.41			
Pathologic tumor stage/nodal status						
T3/N−	1			1		
T4/N−	2.31	1.09–4.92	0.09	1.94	0.91–3.94	0.26
Nx	1.68	0.37–5.37	0.21	1.10	0.31–3.72	0.62
N+	7.39	2.71–19.35	0.01*	4.04	1.30–12.56	0.02*
VATI						
Low VATI	1			1		
High VATI	2.89	0.70–4.32	0.03*	2.47	1.02–4.21	0.04*
Renal dysfunction						
No	1					
Yes	0.58	0.32–1.08	0.08			

*Note*: *Indicates statistical significance.

Abbreviations: BMI, body mass index; CI, confidence interval, ECOG, Eastern Cooperative Oncology Group; GC, gemcitabine plus cisplatin; HR, Hazard ratio; PFS, progression‐free survival; VATI, visceral adipose tissue index.

In the multivariate analysis, nodal status (HR = 4.04, 95% CI 1.30–12.56, *p* = 0.02) and the level of VATI (HR = 2.47, 95% CI 1.02–4.21, *p* = 0.04) were independent prognostic indicators for PFS, as indicated in Table 3. Patients with low VATI demonstrated a 3‐year PFS rate of 60.2%, while those with high VATI exhibited a 3‐year PFS rate of 41.0%.

### 3.3 Chemotherapy‐Related Adverse Events

Most common chemotherapy‐related AEs of any grade and grade ≥ 3 were hematologic toxicities (Table 4). Among them, neutropenia emerged as the most prevalent grade ≥ 3 hematologic AE. The incidence of grade ≥ 3 non‐hematologic AEs in the high‐VATI and low‐VATI groups was 7.2% (*n* = 6) and 13.3% (*n* = 11), respectively. In both groups, the most common non‐hematologic toxicities were nausea and decreased appetite. In the low‐VATI group, three patients (3.6%) experienced grade ≥ 3 fatigue following postoperative GC; however, no patients were observed to develop high‐grade fatigue in the high‐VATI group. A chi‐square test was employed to compare differences between the low‐VATI and the high‐VATI groups. We observed that patients in the low‐VATI group were more likely to experience grade ≥ 3 neutropenia compared to those in the high‐VATI group (*p* = 0.04).

**TABLE 4:** Chemotherapy‐related AEs reported for patients of the high‐ and low‐VATI arms.

Event, *n* (%)	High VATI (*n* = 83)		Low VATI (*n* = 83)	
	All grade	Grade ≥ 3	All grade	Grade ≥ 3
**Hematologic AEs**	75 (90.4)	40 (48.2)	77 (92.8)	51 (61.4)
Neutrophil count decreased	44 (53.0)	27 (32.5)	51 (61.4)	40 (48.2)
Anemia	59 (71.1)	5 (6.0)	57 (68.7)	8 (9.6)
Platelet count decreased	44 (53.0)	8 (9.6)	41 (49.4)	14 (16.9)
WBC decreased	41 (49.4)	16 (19.3)	47 (56.6)	23 (27.7)
**Non‐hematologic AEs**	73 (88.0)	6 (7.2)	77 (92.8)	11 (13.3)
Nausea	60 (72.3)	1 (1.2)	55 (66.3)	2 (2.4)
Decreased appetite	55 (66.3)	0 (0)	50 (60.2)	1 (1.2)
Fatigue	20 (24.1)	0 (0)	28 (33.7)	3 (3.6)
Alopecia	15 (18.1)	0 (0)	20 (24.1)	0 (0)
Pruritus	13 (15.7)	0 (0)	15 (18.1)	0 (0)
Weight decreased	7 (8.4)	0 (0)	12 (14.5)	0 (0)

*Note:* Data are presented as frequency (percentage).

Abbreviations: AEs, adverse events; VATI, visceral adipose tissue index; WBC, white blood cell.

## 4 Discussion

Several cytotoxic chemotherapy agents, including cisplatin, methotrexate, doxorubicin, gemcitabine, and paclitaxel, have demonstrated substantial efficacy as single agents against UC [19]. Notably, in the 1980s, platinum‐based chemotherapy exhibited remarkable responses, and cisplatin‐containing combination therapy has been the first‐line treatment for advanced BC since then [4]. Compared to methotrexate, vinblastine, doxorubicin, and cisplatin (MVAC), the GC regimen has similar survival outcomes and better safety profiles, and thus GC became the standard treatment in advanced BC [20]. The European Association of Urology (EAU) guidelines recommend NAC for T2‐T4aN0M0 BC, and if NAC is not performed, postoperative AC is required [21]. Despite the emergence of numerous novel therapeutic drugs [22, 23], the GC regimen continues to serve as the first‐line treatment for patients with advanced BC.

Today, the global prevalence of obesity, coupled with the increased utilization of cytotoxic drugs in numerous malignancies, underscores the crucial importance of understanding the impact of obesity on cancer patients. A large analysis of the influence of obesity on the pharmacokinetics of cytotoxic drugs has demonstrated a drug‐specific interaction between BMI and pharmacokinetic clearance rates [24]. While BMI is utilized to define obesity, ambiguity persists regarding whether elevated BMI is protective or detrimental to cancer patients [25, 26]. Therefore, a more efficacious approach for measuring obesity is warranted. With the progression of research, investigators have found that adipose distribution may exert a greater impact on cytotoxic clearance than obesity per se, suggesting that individualized dosing based on varying body compositions appears to be a feasible strategy [27, 28], potentially improving outcomes for obese patients without augmenting toxicity. Body composition, a key prognostic indicator in oncology patients, has been extensively investigated [29]. Over the course of the study, it has become evident that BMI, the most commonly employed metric for assessing body composition, fails to differentiate between muscle and adipose tissue and does not provide insight into the distribution of adipose tissue [30]. However, the SAT and VAT are considered to be the main adipose tissues of the human body, and these two different adipose tissues have been shown to exhibit different functions and metabolic characteristics [31].

Currently, there is no consensus regarding the impact of VAT on disease progression and prognosis in cancer patients. Among urological tumors, the study has revealed that for metastatic renal cell carcinoma (mRCC) patients receiving vascular endothelial growth factor (VEGF)‐targeted therapy, higher VAT, rather than BMI, was an independent predictor of improved survival [32]. However, the study conducted by Ladoire et al. yielded contrasting findings; for patients with mRCC receiving VEGF‐targeted therapy, higher visceral adipose area was significantly related to shorter OS [33]. For patients with RCC who received surgical treatment, cause‐specific survival was significantly better in patients with high VAT compared to those with low VAT [34]. At present, the impact of VAT on the prognosis of UC patients is unknown. Researchers have found that high visceral adipose content was a protective factor in patients with advanced UC treated with ICI [13]. In upper tract urothelial carcinoma patients treated with radical nephroureterectomy, the research has shown that the high VAT was associated with better survival outcomes, although it was not statistically significant [12].

In this study, 166 patients with locally advanced BC received postoperative chemotherapy. We have observed that patients in the low‐VATI group exhibited longer PFS compared to those in the high‐VATI group. In the multivariable Cox regression analysis, the level of VATI remained, next to nodal status, a significant risk factor for PFS. One possible explanation is attributed to the multifaceted role of adipose tissue, which, besides its primary function as a fat reservoir, operates as a metabolically and endocrinologically active organ. Among its components, the stromal vascular fraction rich in adipose stromal cells serves as a crucial reservoir of various cytokines and chemokines [35]. Studies indicate that alterations in the expression of these secretory factors may induce mitotic effects across various cancer types [36, 37, 38], thereby enhancing the invasiveness of certain tumors. For instance, plasminogen activator inhibitor 1, produced by adipocytes, endothelial cells, and stromal cells in visceral adipose tissue [39], has been implicated in promoting cell migration and tumor angiogenesis [40], consequently heightening the likelihood of tumor cells transitioning into an invasive phenotype [41, 42]. Regarding chemotherapy‐related AEs, we observed hematologic toxicities as the most common at any grade and grade ≥ 3. Furthermore, we found that patients in the low‐VATI group were more prone to experiencing grade ≥ 3 neutropenia following postoperative GC compared to those in the high‐VATI group. While the precise reasons for this correlation remain elusive, we hypothesize that the VATI level represents a substantially underestimated risk factor in routine clinical practice. Significant implications arise for patients in the high‐VATI group, guiding clinicians to enhance monitoring and increase vigilance. Our next research focus is to quantify VATI more precisely and personalize its administration to patients with BC, aiming to enhance prognosis without increasing toxicity. In cases of patients with low VATI, clinicians should conduct more frequent monitoring of hematological parameters, enabling timely adjustments to treatment plans and interventions. Furthermore, the occurrence of high‐grade hematological AEs can be mitigated in advance by the administration of systemic corticosteroids or cell proliferation‐stimulating agents.

Our study has several limitations. First, due to the retrospective nature and limited sample size of this study, especially in the subgroup analysis, selection bias is inevitable. Second, despite the utilization of PSM to control for known risk factors, the presence of potential unknown confounding variables contributing to bias cannot be entirely dismissed. Third, there are currently no sex‐specific optimal cutoff values for VATI, so multicenter studies are needed to establish clear cutoff values in the future. Fourth, a dynamic analysis of changes in VAT and lifestyle in patients during chemotherapy was not conducted. Therefore, the influence of aerobic exercise and nutritional support on patient prognosis and toxicity reactions remains uncertain at present. Additional prospective studies involving larger cohorts are imperative to validate the findings obtained in our study.

## 5 Conclusions

This study demonstrated that, among patients treated with adjuvant GC chemotherapy for locally advanced BC, patients in the low‐VATI group exhibited a significantly prolonged PFS compared to those in the high‐VATI group. However, no significant difference was observed in terms of OS. Regarding chemotherapy‐related AEs, patients in the high‐VATI group exhibited a relatively lower incidence and severity of toxic reactions.

## Author Contributions

**Zhimin Gao:** conceptualization (lead), data curation (equal), investigation (equal), validation (lead), writing – original draft (lead). **Lei Zhang:** software (equal). **Zhen Li:** formal analysis (equal). **Xu Qin:** project administration (equal). **Zewei Wang:** conceptualization (equal). **Junqi Wang:** funding acquisition (equal). **Nienie Qi:** validation (lead), writing – review and editing (lead). **Hailong Li:** funding acquisition (lead).

## Ethics Statement

This retrospective single‐institutional study was approved by the Ethics Committee of the Affiliated Hospital of Xuzhou Medical University (XYFY2022‐KL340).

## Consent

All patients provided written informed consent. Informed consent to publish was obtained.

## Conflicts of Interest

The authors declare no conflicts of interest.

## Supporting information


Data S1

## Data Availability

The dataset analyzed in this study is available from the corresponding author upon reasonable request.

## References

[1] A. Balar , D. Castellano , P. O'Donnell , et al., “First‐Line Pembrolizumab in Cisplatin‐Ineligible Patients With Locally Advanced and Unresectable or Metastatic Urothelial Cancer (KEYNOTE‐052): A Multicentre, Single‐Arm, Phase 2 Study,” Lancet Oncology 18, no. 11 (2017): 1483–1492. 28967485 10.1016/S1470-2045(17)30616-2

[2] E. Chang , C. Weinstock , L. Zhang , et al., “FDA Approval Summary: Enfortumab Vedotin for Locally Advanced or Metastatic Urothelial Carcinoma,” Clinical Cancer Research 27, no. 4 (2021): 922–927. 32962979 10.1158/1078-0432.CCR-20-2275

[3] T. P. S. Perera , E. Jovcheva , L. Mevellec , et al., “Discovery and Pharmacological Characterization of JNJ‐42756493 (Erdafitinib), a Functionally Selective Small‐Molecule FGFR Family Inhibitor,” Molecular Cancer Therapeutics 16, no. 6 (2017): 1010–1020. 28341788 10.1158/1535-7163.MCT-16-0589

[4] J. Bellmunt and D. P. Petrylak , “New Therapeutic Challenges in Advanced Bladder Cancer,” Seminars in Oncology 39, no. 5 (2012): 598–607. 23040256 10.1053/j.seminoncol.2012.08.007

[5] “Neoadjuvant Chemotherapy in Invasive Bladder Cancer: Update of a Systematic Review and Meta‐Analysis of Individual Patient Data Advanced Bladder Cancer (ABC) Meta‐Analysis Collaboration,” European Urology 48, no. 2 (2005): 202–205, discussion 5‐6. 15939524 10.1016/j.eururo.2005.04.006

[6] M. Galsky , K. Stensland , E. Moshier , et al., “Effectiveness of Adjuvant Chemotherapy for Locally Advanced Bladder Cancer,” Journal of Clinical Oncology 34, no. 8 (2016): 825–832. 26786930 10.1200/JCO.2015.64.1076

[7] M. Louise Catherine , K. Wassim , H. B. Rodney , et al., “Obesity and Complication Risk From Radical Cystectomy: Identifying a Body Mass Index Threshold,” Journal of Urology 209, no. 1 (2022): 111–120. 36250946 10.1097/JU.0000000000002988

[8] D. Yohann , R. Yohann , A. Julien , et al., “Impact of Body Mass Index on the Oncological Outcomes of Patients Treated With Radical Cystectomy for Muscle‐Invasive Bladder Cancer,” World Journal of Urology 35, no. 2 (2016): 229–235. 27272203 10.1007/s00345-016-1852-0

[9] C. Prado , M. Gonzalez , and S. Heymsfield , “Body Composition Phenotypes and Obesity Paradox,” Current Opinion in Clinical Nutrition and Metabolic Care 18, no. 6 (2015): 535–551. 26335310 10.1097/MCO.0000000000000216

[10] M. Prillaman , “Why BMI Is Flawed – and How to Redefine Obesity,” Nature 622, no. 7982 (2023): 232–233. 37903933 10.1038/d41586-023-03257-2

[11] M. Ibrahim , “Subcutaneous and Visceral Adipose Tissue: Structural and Functional Differences,” Obesity Reviews 11, no. 1 (2010): 11–18. 19656312 10.1111/j.1467-789X.2009.00623.x

[12] C. Pickl , S. Engelmann , F. Girtner , et al., “Body Composition as a Comorbidity‐Independent Predictor of Survival Following Nephroureterectomy for Urothelial Cancer of the Upper Urinary Tract,” Cancers 15, no. 2 (2023): 450. 36672398 10.3390/cancers15020450 PMC9857333

[13] D. Martini , J. Shabto , S. Goyal , et al., “Body Composition as an Independent Predictive and Prognostic Biomarker in Advanced Urothelial Carcinoma Patients Treated With Immune Checkpoint Inhibitors,” Oncologist 26, no. 12 (2021): 1017–1025. 34342095 10.1002/onco.13922 PMC8649001

[14] Y. Pan , Z. Chen , L. Yang , et al., “Body Composition Parameters May Be Prognostic Factors in Upper Urinary Tract Urothelial Carcinoma Treated by Radical Nephroureterectomy,” Frontiers in Oncology 11 (2021): 679158, 10.3389/fonc.2021.679158. 34109126 PMC8180865

[15] J. Wang , S. Tan , L. Gianotti , and G. Wu , “Evaluation and Management of Body Composition Changes in Cancer Patients,” Nutrition 114 (2023): 112132. 37441827 10.1016/j.nut.2023.112132

[16] X. Cheng , Y. Zhang , C. Wang , et al., “The Optimal Anatomic Site for a Single Slice to Estimate the Total Volume of Visceral Adipose Tissue by Using the Quantitative Computed Tomography (QCT) in Chinese Population,” European Journal of Clinical Nutrition 72, no. 11 (2018): 1567–1575. 29559725 10.1038/s41430-018-0122-1 PMC6329297

[17] H. Kvist , B. Chowdhury , U. Grangård , U. Tylén , and L. Sjöström , “Total and Visceral Adipose‐Tissue Volumes Derived From Measurements With Computed Tomography in Adult Men and Women: Predictive Equations,” American Journal of Clinical Nutrition 48, no. 6 (1988): 1351–1361. 3202084 10.1093/ajcn/48.6.1351

[18] K. Chua , X. Lin , Y. Wang , Y. Chong , W. Lim , and W. Koh , “Visceral Fat Area Is the Measure of Obesity Best Associated With Mobility Disability in Community Dwelling Oldest‐Old Chinese Adults,” BMC Geriatrics 21, no. 1 (2021): 282. 33910516 10.1186/s12877-021-02226-6 PMC8082923

[19] R. Nadal and J. Bellmunt , “Cytotoxic Chemotherapy for Advanced Bladder and Upper Tract Cancer,” in Bladder Cancer: A Practical Guide, ed. A. M. Kamat and P. C. Black (Springer International Publishing, 2021), 289–304.

[20] H. von der Maase , L. Sengelov , J. Roberts , et al., “Long‐Term Survival Results of a Randomized Trial Comparing Gemcitabine Plus Cisplatin, With Methotrexate, Vinblastine, Doxorubicin, Plus Cisplatin in Patients With Bladder Cancer,” Journal of Clinical Oncology 23, no. 21 (2005): 4602–4608. 16034041 10.1200/JCO.2005.07.757

[21] J. Alfred Witjes , T. Lebret , E. Compérat , et al., “Updated 2016 EAU Guidelines on Muscle‐Invasive and Metastatic Bladder Cancer,” European Urology 71, no. 3 (2017): 462–475. 27375033 10.1016/j.eururo.2016.06.020

[22] M. D. Galsky , J. Á. A. Arija , A. Bamias , et al., “Atezolizumab With or Without Chemotherapy in Metastatic Urothelial Cancer (IMvigor130): A Multicentre, Randomised, Placebo‐Controlled Phase 3 Trial,” Lancet 395, no. 10236 (2020): 1547–1557. 32416780 10.1016/S0140-6736(20)30230-0

[23] J. E. Rosenberg , T. W. Flaig , T. W. Friedlander , et al., “Study EV‐103: Preliminary Durability Results of Enfortumab Vedotin Plus Pembrolizumab for Locally Advanced or Metastatic Urothelial Carcinoma,” Journal of Clinical Oncology 38, no. 6_suppl (2020): 441, 10.1200/JCO.2020.38.6_suppl.441.

[24] S. Alex , C. W. Antonio , H. J. M. Ron , et al., “Evaluation of Alternate Size Descriptors for Dose Calculation of Anticancer Drugs in the Obese,” Journal of Clinical Oncology 25, no. 30 (2007): 4707–4713. 17947717 10.1200/JCO.2007.11.2938

[25] L. M. Jennifer , R. D. Carrie , R. H. Kenneth , et al., “Association of Body‐Mass Index and Outcomes in Patients With Metastatic Melanoma Treated With Targeted Therapy, Immunotherapy, or Chemotherapy: A Retrospective, Multicohort Analysis,” Lancet Oncology 19, no. 3 (2018): 310–322. 29449192 10.1016/S1470-2045(18)30078-0 PMC5840029

[26] J. R. Penelope , J. B. Robin , and R. D. Susan , “Obesity Is Associated With a Poorer Prognosis in Women With Hormone Receptor Positive Breast Cancer,” Maturitas 79, no. 3 (2014): 279–286. 25088248 10.1016/j.maturitas.2014.07.004

[27] J. E. Abraham , L. Hiller , L. Dorling , et al., “A Nested Cohort Study of 6,248 Early Breast Cancer Patients Treated in Neoadjuvant and Adjuvant Chemotherapy Trials Investigating the Prognostic Value of Chemotherapy‐Related Toxicities,” BMC Medicine 13 (2015): 306. 26715442 10.1186/s12916-015-0547-5 PMC4693418

[28] A. L. Wong , K. Y. Seng , E. M. Ong , et al., “Body Fat Composition Impacts the Hematologic Toxicities and Pharmacokinetics of Doxorubicin in Asian Breast Cancer Patients,” Breast Cancer Research and Treatment 144, no. 1 (2014): 143–152. 24481679 10.1007/s10549-014-2843-8

[29] J. Brown , E. Cespedes Feliciano , and B. Caan , “The Evolution of Body Composition in Oncology‐Epidemiology, Clinical Trials, and the Future of Patient Care: Facts and Numbers,” Journal of Cachexia, Sarcopenia and Muscle 9, no. 7 (2018): 1200–1208. 30637983 10.1002/jcsm.12379 PMC6351674

[30] M. Gonzalez , M. Correia , and S. Heymsfield , “A Requiem for BMI in the Clinical Setting,” Current Opinion in Clinical Nutrition and Metabolic Care 20, no. 5 (2017): 314–321. 28768291 10.1097/MCO.0000000000000395

[31] A. Shuster , M. Patlas , J. Pinthus , and M. Mourtzakis , “The Clinical Importance of Visceral Adiposity: A Critical Review of Methods for Visceral Adipose Tissue Analysis,” British Journal of Radiology 85, no. 1009 (2012): 1–10. 21937614 10.1259/bjr/38447238 PMC3473928

[32] S. Steffens , V. Grünwald , K. Ringe , et al., “Does Obesity Influence the Prognosis of Metastatic Renal Cell Carcinoma in Patients Treated With Vascular Endothelial Growth Factor‐Targeted Therapy?,” Oncologist 16, no. 11 (2011): 1565–1571. 22020210 10.1634/theoncologist.2011-0213 PMC3233291

[33] S. Ladoire , F. Bonnetain , M. Gauthier , et al., “Visceral Fat Area as a New Independent Predictive Factor of Survival in Patients With Metastatic Renal Cell Carcinoma Treated With Antiangiogenic Agents,” Oncologist 16, no. 1 (2011): 71–81. 10.1634/theoncologist.2010-0227 PMC3228050 21212435

[34] Y. Naya , S. Zenbutsu , K. Araki , et al., “Influence of Visceral Obesity on Oncologic Outcome in Patients With Renal Cell Carcinoma,” Urologia Internationalis 85, no. 1 (2010): 30–36. 20693825 10.1159/000318988

[35] G. Hajer , T. van Haeften , and F. Visseren , “Adipose Tissue Dysfunction in Obesity, Diabetes, and Vascular Diseases,” European Heart Journal 29, no. 24 (2008): 2959–2971. 18775919 10.1093/eurheartj/ehn387

[36] J. Park , D. Euhus , and P. Scherer , “Paracrine and Endocrine Effects of Adipose Tissue on Cancer Development and Progression,” Endocrine Reviews 32, no. 4 (2011): 550–570. 21642230 10.1210/er.2010-0030 PMC3369575

[37] R. van Kruijsdijk , E. van der Wall , and F. Visseren , “Obesity and Cancer: The Role of Dysfunctional Adipose Tissue,” Cancer Epidemiology, Biomarkers & Prevention 18, no. 10 (2009): 2569–2578. 19755644 10.1158/1055-9965.EPI-09-0372

[38] L. Prantl , F. Muehlberg , N. Navone , et al., “Adipose Tissue‐Derived Stem Cells Promote Prostate Tumor Growth,” Prostate 70, no. 15 (2010): 1709–1715. 20564322 10.1002/pros.21206 PMC4977846

[39] D. Bastelica , P. Morange , B. Berthet , et al., “Stromal Cells Are the Main Plasminogen Activator Inhibitor‐1‐Producing Cells in Human Fat: Evidence of Differences Between Visceral and Subcutaneous Deposits,” Arteriosclerosis, Thrombosis, and Vascular Biology 22, no. 1 (2002): 173–178, 10.1161/hq0102.101552. 11788479

[40] C. Leik , E. Su , P. Nambi , D. Crandall , and D. Lawrence , “Effect of Pharmacologic Plasminogen Activator Inhibitor‐1 Inhibition on Cell Motility and Tumor Angiogenesis,” Journal of Thrombosis and Haemostasis 4, no. 12 (2006): 2710–2715. 17010152 10.1111/j.1538-7836.2006.02244.x

[41] L. Beaulieu , B. Whitley , T. Wiesner , et al., “Breast Cancer and Metabolic Syndrome Linked Through the Plasminogen Activator Inhibitor‐1 Cycle,” BioEssays: News and Reviews in Molecular, Cellular and Developmental Biology 29, no. 10 (2007): 1029–1038. 17876797 10.1002/bies.20640 PMC4046619

[42] N. Hariharan , K. Ashcraft , R. Svatek , et al., “Adipose Tissue‐Secreted Factors Alter Bladder Cancer Cell Migration,” Journal of Obesity 2018 (2018): 9247864. 29887999 10.1155/2018/9247864 PMC5985104

