# Supplementary material for: Visceral Adipose Predicts Prognosis and Toxicities in Locally Advanced Bladder Cancer Patients Treated With Adjuvant Gemcitabine Plus Cisplatin Chemotherapy
Source: Cancer Med. 2025 Mar 27;14(7):e70742. doi: 10.1002/cam4.70742 (PMC11947749; doi:10.1002/cam4.70742)
Supplement: Supplementary file 1 — Data S1 [file CAM4-14-e70742-s001.docx]

**Authorship contribution statement**

Zhimin Gao, serving as the primary author of this paper, played a pivotal role in formulating the overarching research objectives and drafting the initial manuscript. Nienie Qi, on the other hand, contributed significantly by critically reviewing the manuscript.

The collaborative efforts extended to the data collection phase, where Xu Qin and Lei Zhang took the lead in gathering experimental data. Subsequently, Zewei Wang and Zhen Li assumed primary responsibilities for utilizing statistical software to effectively visualize the acquired data, enhancing the clarity of the research findings.

Guiding and overseeing the entire research process were Junqi Wang and Nienie Qi. They provided leadership in the planning and implementation of research activities, ensuring the study's integrity and adherence to established methodologies.

Hailong Li, serving as the corresponding author, played a crucial role in securing financial support for the publication project, underscoring the commitment to disseminating valuable research outcomes.

It is imperative to note that all authors, including those mentioned above, actively participated in the manuscript review process, providing their valuable insights, and have given their consent for the publication of this comprehensive collaborative effort.
